# AAA-ATPase p97 suppresses apoptotic and autophagy-associated cell death in rheumatoid arthritis synovial fibroblasts

**DOI:** 10.18632/oncotarget.11890

**Published:** 2016-09-07

**Authors:** Masaru Kato, Caroline Ospelt, Christoph Kolling, Tomohiro Shimizu, Michihito Kono, Shinsuke Yasuda, Beat A. Michel, Renate E. Gay, Steffen Gay, Kerstin Klein, Tatsuya Atsumi

**Affiliations:** ^1^ Division of Rheumatology, Endocrinology and Nephrology, Hokkaido University Graduate School of Medicine, Sapporo, Japan; ^2^ Center of Experimental Rheumatology, University Hospital Zurich, Zurich, Switzerland; ^3^ Schulthess Clinic, Zurich, Switzerland; ^4^ Department of Orthopaedic Surgery, Hokkaido University Graduate School of Medicine, Sapporo, Japan

**Keywords:** p97, histone deacetylase 6, polyubiquitin, cell death, autophagy

## Abstract

Valosin containing protein (p97) is a chaperone implicated in a large number of biological processes including endoplasmic reticulum (ER)-associated protein degradation and autophagy. Silencing of p97 in rheumatoid arthritis (RA) synovial fibroblasts (RASFs) increased the amount of polyubiquitinated proteins, whereas silencing of its interaction partner histone deacetylase 6 (HDAC6) had no effect. Furthermore, silencing of p97 in RASFs increased not only rates of apoptotic cell death induced by TRAIL but also induced an autophagy-associated cell death during ER stress that was accompanied by the formation of polyubiquitinated protein aggregates and large vacuoles. Finally, we demonstrated an anti-arthritic effect of siRNAs targeting p97 in collagen-induced arthritis in rats. Our data indicate that p97 may be a new potential target in the treatment of RA.

## INTRODUCTION

Rheumatoid arthritis (RA) is characterized by chronic joint inflammation and progressive destruction of cartilage and bone which leads to severe joint pain and ultimately loss of function. Joint-resident RA synovial fibroblasts (RASFs) play a pivotal role in the pathogenesis of RA. RASFs are capable of producing a large set of inflammatory cytokines, chemokines and matrix-degrading enzymes and thereby actively contribute to the inflammatory and joint destructive state in RA [1]. Since RASFs produce large amounts of proteins it was estimated that as much as 30% of newly synthesized proteins are misfolded [2]. The continuous removal of misfolded proteins is therefore essential for cell homeostasis.

The ATPase valosin containing protein (p97/VCP) is a chaperone implicated in a large number of biological processes including endoplasmic reticulum (ER) associated degradation [3], macroautophagy (hereafter referred to as autophagy) [4], and cell cycle regulation [5]. Owing to its role in protein quality control and cell survival, p97 has also been implicated as a potential therapeutic target in cancer [5, 6]. Histone deacetylase 6 (HDAC6) is an unique member of HDACs since it is not only present in the nucleus but it is also localized in the cytoplasm where it deacetylases non-histone substrates such as microtubules [7]. Also HDAC6 contributes to the cellular protein quality control by binding polyubiquitinated misfolded proteins, modifying the dynamics of microtubules, and finally delivering the misfolded proteins along the microtubules to autophagosomes for degradation [8]. p97 and HDAC6 have been shown to interact with each other [9, 10] and a finely tuned balance of these proteins was suggested to determine the fate of misfolded proteins in embryonic and 3T3 fibroblasts. An imbalance of a p97-HDAC6 molar ratio in favor of HDAC6 was described to enhance the formation of ubiquitin protein aggregates, whereas p97 promotes protein degradation [9]. Here, we describe the role of a p97-controlled polyubiquitin turnover in cell death pathways in RASFs and the beneficial effects of p97 inhibition in an *in vivo* arthritis model.

## RESULTS

### The expression of p97 and the expression of HDAC6 in RASFs and OASFs

**Figure 1:**
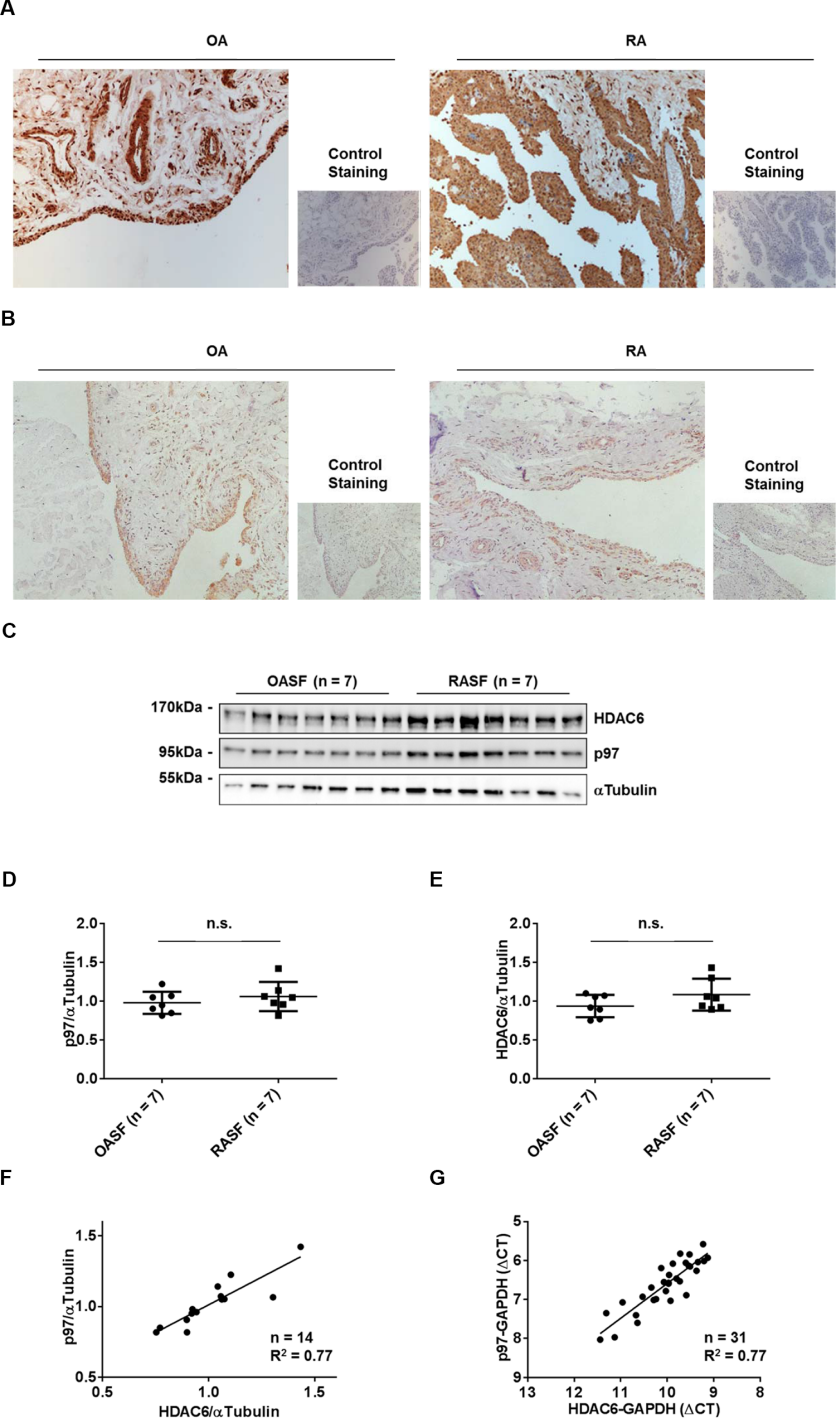
Expression of p97 and HDAC6 in synovial tissues and synovial fibroblasts Representative staining of synovial tissues from OA and RA patients with anti-p97 antibodies (**A**) and anti-HDAC6 antibodies (**B**). Original magnification ×100. Expression of p97 and HDAC6 in OASFs and RASFs, as determined by Western blotting (**C**). Quantification of Western blot results (**D**, **E**). Values are the mean ± SD. Correlation of p97 and HDAC6 expression at protein levels (**F**), as determined by Western blotting, and mRNA levels (**G**), as determined by quantitative reverse transcription–polymerase chain reaction analysis.

Since both p97 and HDAC6 control the fate of misfolded proteins [9], we first evaluated the expression levels of p97 and HDAC6 by immunohistochemistry in synovial tissues obtained from RA and osteoarthritis (OA) patients, as well as by Western blotting and Real-time PCR in cultured synovial fibroblasts (RASFs and OASFs). Staining of p97 and HDAC6 was restricted to the lining layer and vessels of synovial tissues and was similarly detected in RA and OA patients (Figure 1A, 1B). Consistent with this, p97 and HDAC6 proteins in cultured synovial fibroblasts reached equal levels in RA and OA patients (Figure 1C–1E). Interestingly, the expression levels of p97 and HDAC6 in RASFs and OASFs positively correlated at both protein (Figure 1F) and mRNA (Figure 1G) levels, suggesting the presence of a co-regulating factor.

### p97, HDAC6 and polyubiquitinated protein interactions in RASFs

**Figure 2:**
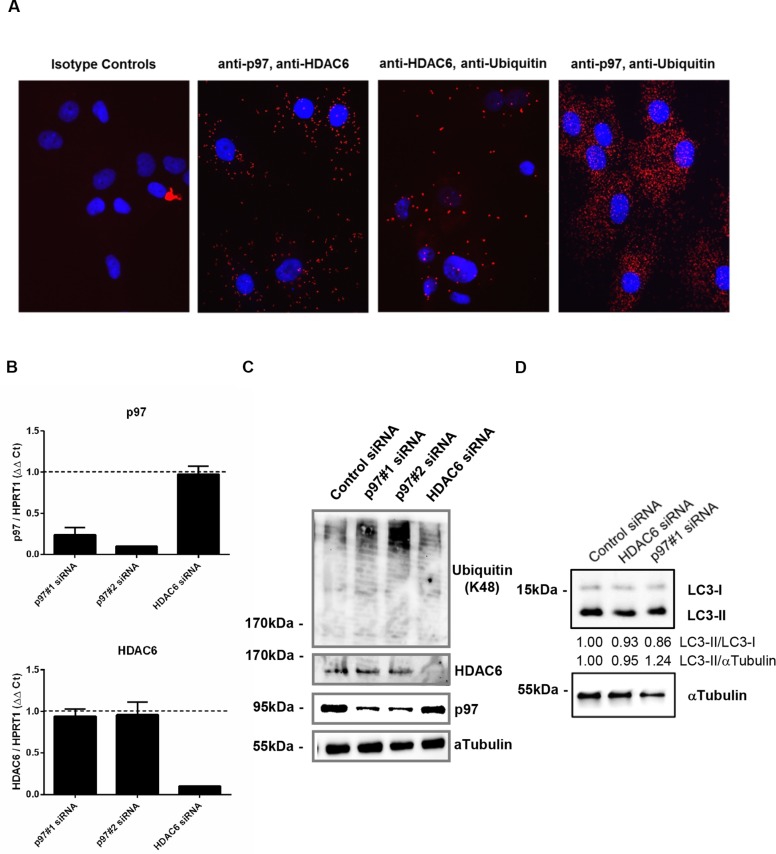
Intracellular interaction among p97, HDAC6 and polyubiquitinated proteins and the effect of silencing of p97 or HDAC6 on the expression of their interacting partners in RASFs Cells were fixed, incubated with anti-p97, anti-HDAC6 and anti-polyubiquitin (K48) antibodies and applied to *in situ* proximity ligation assay (red), followed by DAPI staining (blue) (**A**). Cells were transfected with siRNAs targeting p97 (p97#1, p97#2), HDAC6 or control siRNAs (0.5 μM siRNA to 2.5 × 10^5^ cells). Expression levels of p97 and HDAC6 mRNA were analyzed 48 hours after transfection by quantitative Real-time PCR using HPRT1 as endogenous control (**B**). Expression levels of polyubiquitinated (K48 conjugated) proteins, HDAC6, and p97 (**C**) and the induction of autophagy monitored by LC3-II/I conversion (**D**) 48 hours after the transfection were determined by Western blotting. Expression levels of α-tubulin were used as endogenous control.

Having observed that p97 and HDAC6 levels are also balanced in RASFs as it was previously shown for other fibroblast types [9], we next evaluated the interaction between p97, HDAC6 and polyubiquitinated proteins. Using proximity ligation assays, we detected intracellular interactions of p97 with HDAC6, HDAC6 with polyubiquitin and p97 with polyubiquitin in RASFs (Figure 2A). The siRNA-mediated knockdown of p97 did not affect HDAC6 mRNA and protein levels, and vice versa silencing of HDAC6 did not affect levels of p97 (Figure 2B, 2C). Silencing of p97 in RASFs induced the accumulation of lysine 48 (K48)-conjugated (Figure 2C) but not lysine 63 (K63)-conjugated polyubiquitinated proteins (data not shown). On the other hand, silencing of HDAC6 did not alter levels of polyubiquitinated proteins in RASFs. Knockdown of p97 or HDAC6 did not affect the induction of autophagy monitored by LC3 conversion (Figure 2D). Since interfering with p97 expression levels in RASFs was sufficient to increase levels of protein poly-ubiquitination without further stimulation, we concentrated in the following experiments on the function of p97 in RASFs.

### p97 protects RASF from TRAIL-induced apoptotic cell death

**Figure 3:**
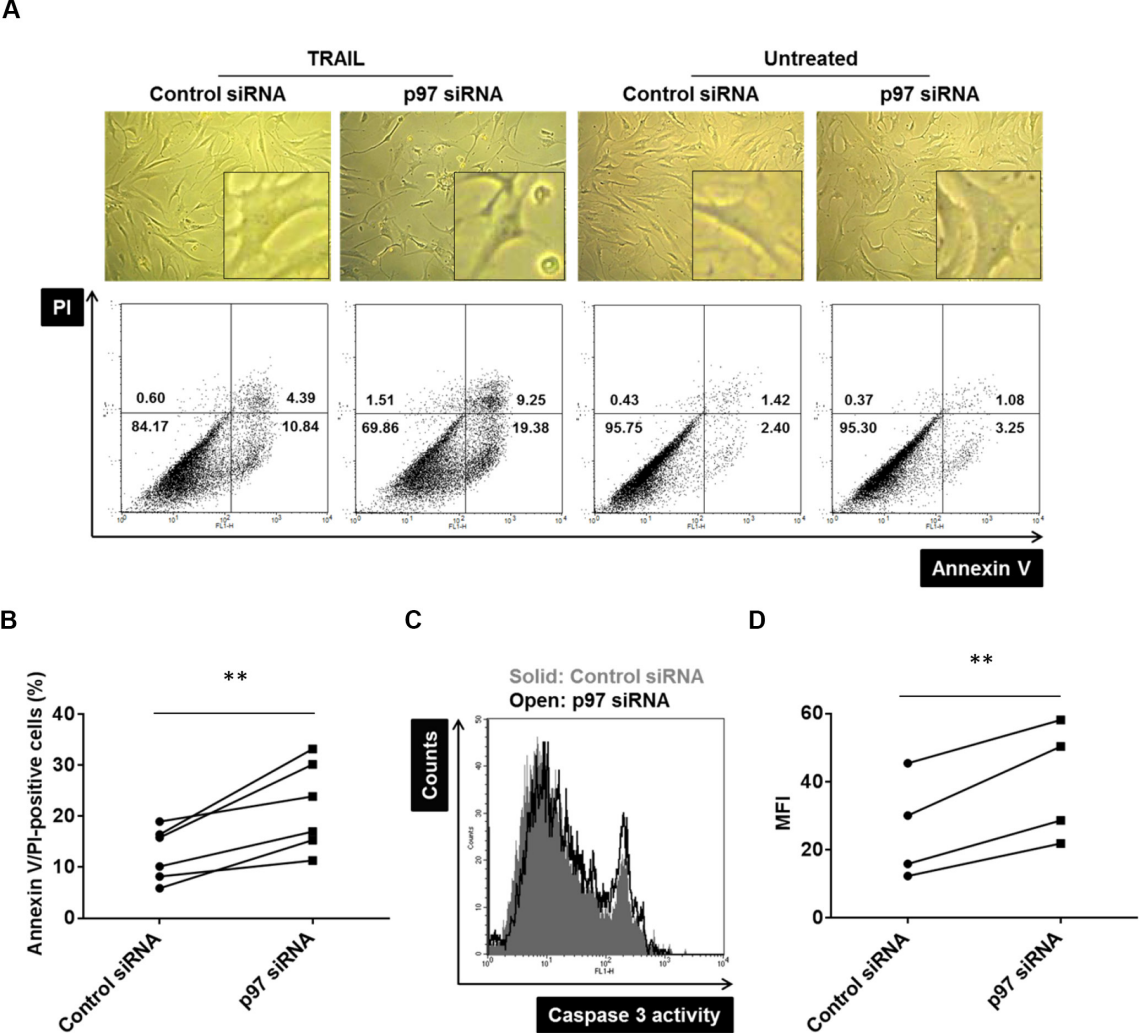
The protective role of p97 in TRAIL-induced apoptosis in RASFs Cells were transfected with siRNAs targeting p97 or control siRNAs (0.5 μM siRNA to 2.5 × 10^5^ cells) and treated with 100 ng/ml TRAIL for 24 hours. Dead cells were evaluated microscopically (original magnification ×100), by flow cytometry using annexin V/propidium iodide (PI) staining (**A**, **B**), and by a caspase-3 activity assay (**C**, **D**). ** = *p* < 0.01.

We have previously shown that the accumulation of polyubiquitinated proteins in RASFs leads to the induction of cell death pathways [11]. Given that p97 plays a critical role in polyubiquitin turnover, we hypothesized that p97 might have a protective role in induction of cell death pathways in RASFs. The siRNA mediated silencing of p97 increased TRAIL-induced annexin V- or PI-positive cells by 74 ± 4% (mean ± SD) compared to control siRNA transfected cells (Figure 3A, 3B). This was accompanied by caspase-3 activation (Figure 3C, 3D), indicating a protective role of p97 against apoptotic cell death in RASFs. Knockdown of p97 did not increase spontaneous annexin V- or PI-positive RASFs (Figure 3A).

### p97 protects RASFs from ER stress-induced autophagy-associated cell death

**Figure 4:**
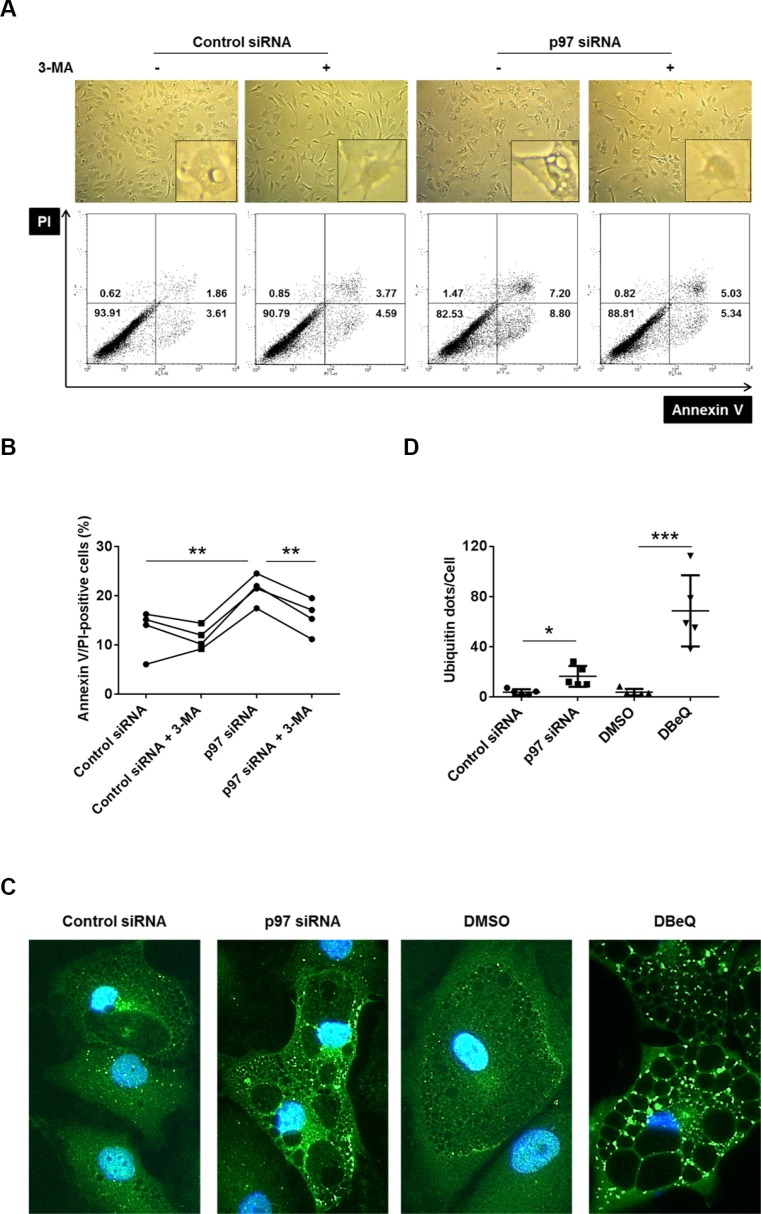
Effects of p97 inhibition on cell death and formation of polyubiquitinated protein aggregates and vacuoles in RASFs under conditions of endoplasmic reticulum stress Cells were transfected with siRNA targeting p97 or control siRNA (0.5 μM siRNA to 2.5 × 10^5^ cells) and then treated for 72 hours with 5 μM thapsigargin (TG) in presence or absence of 5 mM 3-methyladenine (3-MA). Dead cells were evaluated microscopically (original magnification x100) and by flow cytometry using annexin V/propidium iodide (PI) staining. Numbers in each compartment are the percentage of cells (**A**, **B**). Cells were treated as above (in absence of 3-MA), fixed and stained with anti-ubiquitin antibodies (green) and DAPI (blue). Original magnification ×400 (**C**). Ubiquitin-positive dots per cell were calculated with a software (**D**). * = *p* < 0.05; ** = *p* < 0.01; *** = *p* < 0.001.

We have recently described that RASFs are, compared to OASFs, hypersensitive to a non-apoptotic, autophagy-associated cell death under conditions of severe ER stress, leading to a massive cytoplasmic vacuolization and the formation of polyubiquitinated protein aggregates [11]. In order to analyze a potential role of p97 in an autophagy-associated cell death induction in RASFs, we inhibited p97 in RASFs by siRNA transfection or treatment with the p97 inhibitor DBeQ, followed by a treatment with the ER stress inducer TG (5 μM), in the presence or absence of the autophagy inhibitor 3-MA. Both silencing (Figure 4A, 4B) and inhibition of p97 by DBeQ treatment (Supplementary Figure S1A, S1B) enhanced the cytoplasmic vacuolization and increased the amount of annexin V- or PI-positive cells. These effects were inhibited by treatment with the autophagy inhibitor 3-MA (Figure 4A, 4B, Supplementary Figure S1A, S1B). Furthermore, silencing and inhibition of p97 resulted in increased formation of ER-stress-induced polyubiquitinated protein aggregates (Figure 4C, 4D), and 3-MA treatment reduced the intensity of polyubiquitin staining in DBeQ treated cells during ER stress (Supplementary Figure S1C). These results point to a protective role of p97, not only against an apoptotic cell death but also against the autophagy-associated cell death in RASFs. Since 5 μM of TG used in previous experiments induces very severe ER stress in RASFs [11], we also evaluated the role of p97 under conditions of a less severe ER stress induced by smaller doses of TG (5 to 500 nM). Treatment of RASFs with the p97 inhibitor DBeQ and TG concentrations as low as 50 nM were sufficient to induce a cytoplasmic vacuolization (Supplementary Figure S1D) and the formation of polyubiquitinated protein aggregates (Supplementary Figure S1E, S1F). These effects were not observed in the absence of DBeQ. These results indicate that the inhibition of p97 boosts ER stress-induced cell damage from tolerable to intolerable levels in RASFs.

### The role of p97 in arthritis and proliferation of synovial fibroblasts *in vivo*

**Figure 5:**
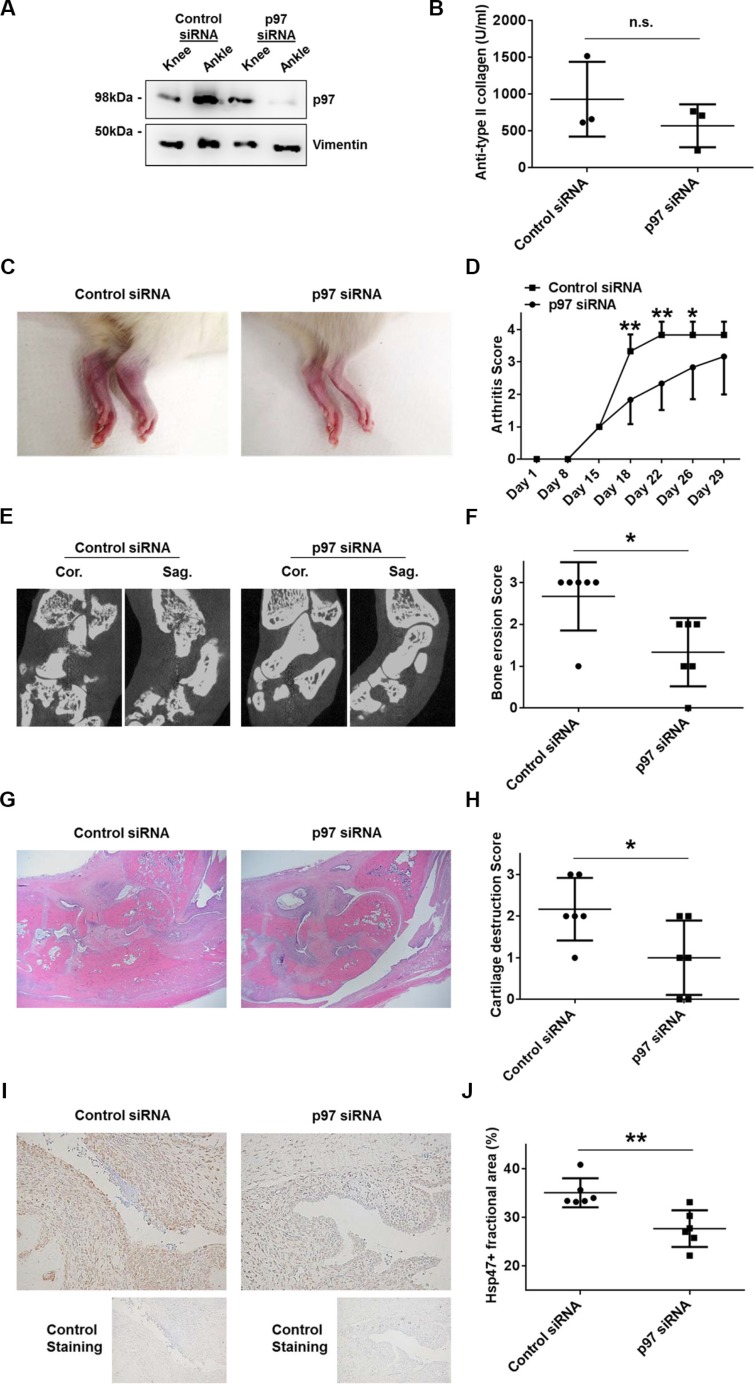
Effects of p97 inhibition in an *in vivo* arthritis model CIA was induced in 7-week-old Lewis rats. Scrambled or p97 siRNA (10 μM)-atelocollagen complexes were injected into ankle joints of rats 14 days after the first immunization with type II collagen (day 15). The siRNA-mediated knockdown of p97 in collagen-induced arthritis (CIA) rat synovial tissues obtained from knee and ankle joints three days after the injection of siRNA into ankle joints was verified by Western blotting (**A**). Serum levels of antibodies against type II collagen on the day of sacrifice (day 29) (**B**). Representative pictures of the ankle joints on day 22 (**C**). Arthritis score following the induction of CIA (**D**). Bone erosion was assessed by micro-CT on day 29. Cor., coronal; Sag., sagittal. (**E**, **F**). Hematoxylin and Eosin staining of the ankle joints (**G**). Cartilage destruction was quantified histologically (**H**). Hsp47 was labelled to evaluate the proliferation of fibroblasts in synovial tissues. Original magnification ×100. Values are the mean ± SD (**I**, **J**). * = *p* < 0.05; ** = *p* < 0.01.

In order to evaluate the role of p97 in synovial tissues *in vivo*, we induced collagen-induced arthritis (CIA) in Lewis rats followed by an intra-articular injection of siRNAs targeting p97 or control siRNAs. To evaluate proliferation rates of synovial fibroblasts, rat synovial tissues were immunolabeled with Hsp47 [12]. The efficiency of p97 siRNAs was evaluated in CIA tissues obtained from knee and ankle joints three days after the injection of siRNAs into ankle joints. Western blotting revealed a sufficient knockdown of p97 in ankle joints, whereas p97 levels in non-injected knees were not affected (Figure 5A). Serum levels of antibodies against type II collagen were similar in the control siRNA group and the p97 siRNA group (Figure 5B). The intra-articular injection of p97 siRNAs, compared to control siRNAs, significantly suppressed arthritis scores (Figure 5C, 5D), grades of bone erosion (Figure 5E, 5F) and cartilage destruction (Figure 5G, 5H), and the Hsp47-positive fractional area in synovial tissues (Figure 5I, 5J) of ankle joints.

## DISCUSSION

Continuous removal of misfolded proteins by the ubiquitin-proteasome system and the autophagy-lysosome pathway is essential for the survival of cells [13]. A key function of p97 is to maintain protein homeostasis through a network of protein quality processes. A large collection of p97-interacting proteins has been identified through proteomic studies [14]. Among those, HDAC6 was described to not only interact with p97 but also to interact with polyubiquitinated chains [9]. We were able to verify that these interactions also occur in RASFs. Both, p97 and HDAC6 play an essential role in the removal of misfolded proteins. Whereas p97 is thought to facilitate the ubiquitin-proteasome dependent degradation of misfolded proteins, HDAC6 delivers them along microtubules to autophagosomes for degradation. Beside the direct interaction of p97 and HDAC6 a counteracting role of these molecules was described interfering with the ability of their partner to interact with ubiquitin [9]. Consistent with previous reports in other fibroblasts [9], we showed a fine balance between p97 and HDAC6 expression levels in RASFs. We identified p97 as a critical component in controlling the polyubiquitin turnover in RASFs. Whereas silencing of p97 in RASFs was sufficient to increase levels of polyubiquitinated proteins without a further stimulation, silencing of HDAC6 had no effect. To date, few studies have shown the efficacy of HDAC6 inhibitors in arthritis models *in vivo* [15, 16], however, the effects are likely not related to the HDAC6 function in protein turnover.

p97 was shown to have both pro- and anti-apoptotic roles but its role in synovial tissues has not been investigated yet. On the one hand, p97 is involved in the processing of caspase-9 [17]. Conversely, several pro-apoptotic genes are upregulated following knockdown of p97 [18]. In addition, p97 activates NFκB by dissociating its inhibitor IκBα, resulting in increased expression levels of anti-apoptotic genes [19]. An impaired ER-associated protein degradation caused by p97 inhibition can also trigger apoptosis [20]. The anti-apoptotic roles of p97 are most relevant for the cell loss in inclusion body myopathy with Paget's disease of bone and frontotemporal dementia which is caused by mutations in the p97 gene [21]. In RA, the apoptosis-resistant phenotype of RASFs has been linked closely to the progressive destruction to articular cartilage and hallmarks synovial cell activation and contributes to chronic inflammation and hyperplasia [22]. A number of molecules have been shown to account for the apoptosis-resistant phenotype of RASFs [23–30] and our data indicate that also p97 fulfills a protective role against a TRAIL-induced apoptotic cell death in RASFs. Our immunohistochemical analysis showed not only high levels of p97 in the synovial lining but also significant expression levels of p97 in vessels of synovial tissues. High endothelial levels of p97 were previously also shown in diverse types of cancers [31], however, the endothelial function of p97 has not been studied yet. A proteomic study on cultured, apoptotic human umbilical vein endothelial cells identified decreased expression levels of p97 and suggested an anti-apoptotic role of p97 also in the vascular endothelium [32]. The p97 inhibitor DBeQ was shown to rapidly activate caspase-3/7 in HeLa cells but it is not clear whether this is a specific effect of p97 inhibition or an off-target effect of this compound [33]. In our study, the treatment of RASFs with DBeQ resulted in increased cell death rates (data not shown), while the siRNA-mediated knockdown of p97 did not increase spontaneous cell death (Figure 3A), indicating an off-target pro-apoptotic effect of DBeQ in RASFs.

Beside the anti-apoptotic role of p97 in RASFs, we also identified a protective role of p97 in an autophagy-associated cell death in these cells. Although autophagy constitutes a cytoprotective response activated by cells to cope with stress and is rather protective of cell death, induction of autophagy also leads to cell death under certain conditions [34]. Autophagy-associated cell death, also called autophagic cell death, is defined as cell death that is accompanied by a massive cytoplasmic vacuolization and which can be suppressed by the inhibition of the autophagic pathway by chemicals or genetic means [35]. We have previously described a hypersensitivity to autophagy-associated cell death under conditions of severe ER stress induced by 5 μM TG in RASFs compared to OASFs [11]. The precise molecular mechanisms regulating this autophagy-associated cell death in RASFs are incompletely understood. p97 has been shown to play an essential role in the maturation of autophagosomes in the late stage of autophagy [4, 36] and an impaired maturation of autophagosome might activate autophagy-associated cell death. In RASFs, the inhibition of autophagy by 3-MA reduced levels of cell death induced by p97 silencing and inhibition.

Molecular targets interfering with cell death pathways are potential therapeutic targets in cancer and p97 inhibitors have promising anti-cancer effects [14]. Our data provide evidence for beneficial effects of interfering with cell death pathways in RASFs and in CIA *in vivo* by the inhibition of p97. p97 exhibited a critical role compared to HDAC6 in terms of promoting the polyubiquitin turnover in RASFs. The inhibition of p97 promoted not only a TRAIL-induced apoptotic cell death but also the ER stress-induced autophagy-associated cell death in RASFs and suppressed CIA and proliferation of synovial fibroblasts *in vivo*. p97, as in the treatment of cancer, is expected to be a potential therapeutic target for RA through the regulation of polyubiquitin turnover and cell death pathways in synovial fibroblasts, key players in the pathogenesis of RA.

## MATERIALS AND METHODS

### Patient samples and cell preparation

Synovial fibroblasts were derived from synovial tissue specimens that were obtained from RA and OA patients during joint replacement surgery (Department of Orthopedic Surgery, Schulthess Clinic, Zurich, Switzerland). All RA patients fulfilled the American College of Rheumatology criteria for classification of the disease [37] and all patients provided informed consent. Cells were cultured as described elsewhere [38] and used between passages 4 to 8 for all experiments.

### Immunohistochemistry for human synovial tissues

After deparaffinization, tissue sections of RA and OA patients were pre-treated with citrate buffer (10 mM sodium citrate, pH 6.0). Endogenous peroxidase activity was disrupted with 3% H_2_O_2_. Nonspecific protein binding was blocked with 1% bovine serum albumin/ 5% goat serum for 40 minutes. Mouse monoclonal anti-p97 antibodies (Abcam), mouse monoclonal anti-HDAC6 antibodies (Santa Cruz) or mouse IgG2a (isotype control) were applied over night at 4°C. Slides were washed in PBS-T (0.05% Tween 20 in PBS) and incubated with biotinylated goat anti-mouse antibodies (1:1000; Jackson ImmunoResearch). The signal was amplified with ABC reagent (Vector Laboratories) and detected with 3,3′-diaminobenzidine (Vector Laboratories).

### Treatment of RASFs

To induce cell death, cells were treated with 100 ng/ml tumor necrosis factor (TNF)-related apoptosis-inducing ligand (TRAIL; R&D Systems) for 24 hours or 5 nM to 5 μM of the ER stress inducer thapsigargin (TG; Enzo Life Sciences) for 72 hours in the presence or absence of 5 mM of the autophagy inhibitor 3-methyladenine (3-MA; Enzo Life Sciences). Where indicated, cells were treated with 5 μM of the selective p97 inhibitor N^2^, N^4^-dibenzylquinazoline-2,4-diamine (DBeQ; Sigma-Aldrich) [33]. Controls were treated with matched amounts of DMSO.

### Transfection of RASFs

2.5 × 10^5^ cells were transfected with 0.5 μM siRNA targeting p97 (Qiagen) or HDAC6 (Qiagen) or scrambled siRNAs as a control using the Amaxa Basic Nucleofector Kit for Primary Mammalian Fibroblasts (Lonza). 48 hours after transfection cells were treated as indicated and then harvested for Western blotting, RNA isolation or flow cytometry. Knockdown of p97 and HDAC6 was verified by Western blotting.

### Western blotting

Cells were lysed in Laemmli buffer (62.5 mM TrisHCl, 2% SDS, 10% Glycerol, 0.1% Bromphenolblue, 5 mM β-mercaptoethanol). Whole cell lysates were separated on 10% SDS polyacrylamide gels and electro blotted onto nitrocellulose membranes (Whatman). Membranes were blocked for 1 hour in 5% (w/v) non-fat milk in TBS-T (20 mM Tris base, 137 mM sodium chloride, 0.1% Tween 20, pH 7.6). The membranes were probed with antibodies against p97 (Abcam), HDAC6 (Cell Signaling), ubiquitin Lys48 (Merck), ubiquitin Lys63 (Millipore), LC3B (Cell Signaling) or α-tubulin (Abcam) as an endogenous control. As secondary antibodies, horseradish peroxidase-conjugated goat anti-rabbit or goat anti-mouse antibodies (Jackson ImmunoResearch) were used. Signals were detected using the ECL Western blotting detection reagents (GE Healthcare) and the Alpha Imager Software system (Alpha Innotech). Expression analysis of specific proteins was performed by pixel quantification of the electronic image.

### Real-time polymerase chain reaction (PCR)

Total RNA was isolated from cells using the ReliaPrep RNA Cell Miniprep System (Promega) including on column DNase I (Promega) digest and reversed transcribed. Real-time PCR was performed using SYBR green (Applied Biosystems) and primers specific for p97 and HDAC6. The primer sequences were as follows: p97 forward, 5′- AGCTGCTCACCATGTGGTTTGGG-3′; p97 reverse, 5′- CAGCTTGGCGGGCCTTGTCA-3′; HDAC6 forward, 5′- GAAAGTCACCTCGGCATCAT-3′; HDAC6 reverse, 5′- TAGTCTGGCCTGGAGTGGAC-3′. Constitutively expressed human glyceraldehyde 3-phosphate dehydrogenase (GAPDH) and hypoxanthin guanin phosphoribosyltransferase (HPRT1) were measured for internal standard sample normalization using primers as described elsewhere [39]. Relative mRNA expression levels were calculated by the comparative threshold cycle method (ΔCt and ΔΔCt).

### Analysis of cell death

After treatment, cells were detached with trypsin, washed twice with PBS, and resuspended in annexin V binding buffer (BD Biosciences) at a concentration of 1 × 10^6^ cells/ ml. Next, cells were incubated with FITC annexin V (BD Biosciences) and propidium iodide (PI, Sigma-Aldrich) for 15 minutes at room temperature in the dark, and analyzed by flow cytometry (FACSCalibur, BD Biosciences).

### Analysis of caspase-3 activity

Following treatment, cells were detached with trypsin, resuspended in cell culture medium at a concentration of 1 × 10^6^ cells/ ml and incubated with caspase-3 substrate (NucView 488, Biotium) for 20 minutes at room temperature in the dark. Caspase-3 activity was analyzed by flow cytometry (FACSCalibur).

### Immunocytochemistry

Cells were cultured in chamber slides (Lab-Tek, Nunc), fixed with 4% paraformaldehyde and permeabilized with TBS containing 0.1% Triton X-100. Non-specific binding was blocked with 1% bovine serum albumin/ 5% goat serum for 40 minutes. Slides were incubated with anti-ubiquitin Lys48 antibodies overnight at 4°C. After washing, slides were incubated with FITC–conjugated secondary antibodies (Thermo Scientific) for 30 minutes and nuclei were stained with DAPI (Sigma-Aldrich). Slides were covered with fluorescent mounting medium (Dako) and analyzed with a fluorescence microscope (Axio Imager, Carl Zeiss). Ubiquitin-positive dots per cell were calculated with a software (Hybrid Cell Count, Keyence).

### Proximity ligation assays

Intracellular protein interactions were analyzed using the Duolink^®^ kit (Olink Bioscience) which is based on the use of two unique and bi-functional probes called PLA™. Each probe consists of a secondary antibody attached to a unique synthetic oligonucleotide that acts as a reporter. Cells were cultured in chamber slides (Lab-Tek), fixed with 4% paraformaldehyde and permeabilized with TBS containing 0.1% Triton X-100. Non-specific binding was blocked with 1% bovine serum albumin/ 5% goat serum for 40 minutes. Slides were incubated with two primary antibodies overnight at 4°C. After washing, slides were incubated with the secondary oligonucleotide-linked antibodies provided in the kit. The oligonucleotides bound to the antibodies were hybridized, ligated, amplified, and detected using a fluorescent probe provided in the kit and nuclei were stained with DAPI. Slides were covered with fluorescent mounting medium and analyzed with a fluorescence microscope (Axio Imager).

### Induction of collagen-induced arthritis (CIA) and administration of siRNA in rats

CIA was induced in six 7-week-old female Lewis rats by immunizing 200 ml solution of 1 mg/ml porcine type II collagen (Chondrex) dissolved in 0.05 M acetic acid and emulsified in incomplete Freund's adjuvant (Chondrex) at the base of the tail [40]. Seven days later, the rats received a booster immunization with the collagen. On day 15, the rats received an intra-articular injection, into both right and left ankles, of a 50 μl solution containing 10 μM siRNA–atelocollagen (Koken) complexes targeting p97 (Qiagen) or control. Silencing efficiency of p97 siRNAs was evaluated by Western blotting in in CIA tissues obtained from knee and ankle joints three days after the injection of siRNAs into ankle joints. On days 1, 8, 15, 18, 22, 26 and 29, arthritis was scored according to paw thickness and ankle diameter from 0 (neither erythema nor swelling) to 4 (erythema and severe swelling that encompassed the ankle, foot, and digits or ankylosis of the limb) [41]. Bone erosion of each ankle joint was scored in a blinded manner on day 29 by micro–computed tomography (micro-CT) (R_mCT2, Rigaku) from 0 (normal joint) to 3 (severe cartilage and bone erosions) [42]. The experimental protocol was approved by the Animal Ethics Committee at Hokkaido University.

### ELISA for anti-type II collagen antibodies in rat sera

Blood samples were collected from rats on day 29. Serum level of anti-type II collagen IgG was detected by enzyme-linked immunosorbent assay (Chondrex).

### Immunohistochemistry for rat synovial tissues

Rats were sacrificed on day 29 for immunohistochemical analyses. The ankles were decalcified and embedded in paraffin. After deparaffinization, the tissue sections were pre-treated with citrate buffer (pH 6.0) for 40 minutes at 80°C. Endogenous peroxidase activity was disrupted with 0.3% H_2_O_2_ in methanol. Nonspecific protein binding was blocked with G-Block (Genostaff) for 10 minutes. Endogenous biotin, biotin receptors, and avidin binding sites were blocked with the avidin/biotin blocking kit (SP-2001, Vector Laboratories). Mouse monoclonal anti-Hsp47 antibodies (Enzo Life Sciences) and mouse IgG2b (isotype control) were applied (2 μg/ml) over night at 4°C. Slides were washed in TBS-T and incubated with biotinylated goat anti-mouse antibodies (Dako). The signal was amplified with horseradish peroxidase-conjugated streptavidin (Nichirei Bioscience) and detected with 3,3′-diaminobenzidine. Cartilage destruction was scored from 0 (no cartilage loss) to 3 (complete loss of articular cartilage) [43]. The Hsp47-positive lining area was adjusted to the linear horizontal length (μm^2^/μm) of the analyzed lining [12].

### Statistical analysis

Mean ± SD values were calculated. Unpaired or paired *t-test*s were used for statistical evaluation of the data using GraphPad Prism 5.0. *P value*s less than 0.05 were considered significant. The fit of regression model was assessed by coefficient of determination.

## SUPPLEMENTARY MATERIALS FIGURES


