# Supplementary material for: AAA-ATPase p97 suppresses apoptotic and autophagy-associated cell death in rheumatoid arthritis synovial fibroblasts
Source: Oncotarget. 2016 Sep 7;7(39):64221–32. doi: 10.18632/oncotarget.11890 (PMC5325437; doi:10.18632/oncotarget.11890)
Supplement: Supplementary file 1 [file oncotarget-07-64221-s001.pdf]

AAA-ATPase p97 suppresses apoptotic and autophagy-associated cell death in rheumatoid arthritis synovial fibroblasts

Supplementary Materials

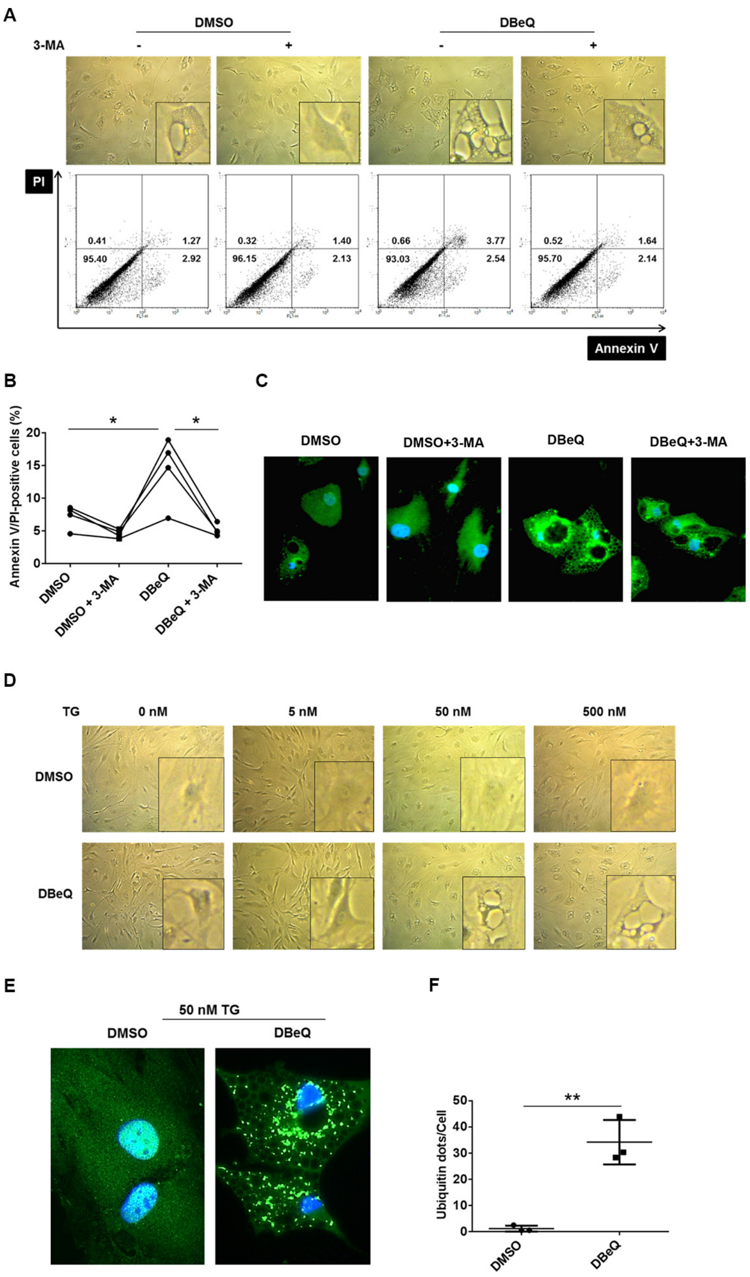

Supplementary Figure S1: Effects of p97 inhibition on cell death and formation of polyubiquitinated protein aggregates and vacuoles in RASFs under conditions of endoplasmic reticulum stress. Cells were treated with 5 µM DBeQ or DMSO and then treated for 72 hours with 5 µM thapsigargin (TG) in presence or absence of 5 mM 3-methyladenine (3-MA). Dead cells were evaluated microscopically (Original magnification ×100) and by flow cytometry using annexin V/propidium iodide (PI) staining. Numbers in each compartment are the percentage of cells (**A**, **B**). Cells were treated as above, fixed and stained with anti-ubiquitin antibodies (green) and DAPI (blue). Original magnification ×200 (**C**). Cells were treated for 72 hours with 5 to 500 nM TG in presence or absence of 5 µM DBeQ. Vacuoles were evaluated microscopically. Original magnification ×100 (**D**). Cells were treated as above, fixed and stained with anti-ubiquitin antibodies (green) and DAPI (blue). Original magnification ×400 (**E**). Ubiquitin-positive dots per cell were calculated with a software (**F**). * = $p < 0.05$; ** = $p < 0.01$.
